# Clinical–Ultrasound Model to Predict the Clinical Course in Bronchiolitis

**DOI:** 10.3390/children11080987

**Published:** 2024-08-14

**Authors:** Lucía Rodríguez García, Elena Hierro Delgado, Ignacio Oulego Erroz, Corsino Rey Galán, Juan Mayordomo Colunga

**Affiliations:** 1 Childhood and Adolescence Clinical Management Area, Hospital Universitario Central de Asturias, 33011 Oviedo, Spain; crey@uniovi.es (C.R.G.);; 2 Paediatrics Department, Complejo Asistencial Universitario de León, 24071 León, Spain; 3 Institute of Biomedicine of León, Universidad de León, 24071 León, Spain; 4 Cooperative Research Networks Oriented to Health Outcomes (RICORS), Instituto de Salud Carlos III, RD21/0012/0020, 28029 Madrid, Spain; 5 Department of Medicine, Universidad de Oviedo, 33006 Oviedo, Spain; 6 Health Research Institute of the Principality of Asturias (ISPA), 33011 Oviedo, Spain; 7 Biomedical Research Networking Center (CIBER)-Respiratory Diseases, Instituto de Salud Carlos III, 28029 Madrid, Spain

**Keywords:** bronchiolitis, ultrasound, hospitalization, oxygen inhalation therapy, prognosis

## Abstract

Background: The aim of the present study was to develop a clinical–ultrasound model for early detection of hospital admission, pediatric intensive care unit (PICU) admission, and oxygen requirement in children diagnosed with acute bronchiolitis (AB). Furthermore, the prognostic ability of models including sonographic data from antero-lateral, lateral-posterior, and posterior areas (eight zones) vs. antero-lateral and lateral-posterior areas (six zones) vs. only antero-lateral areas (four zones) was analyzed. Methods: A prospective study was conducted on infants under 12 months with AB. A lung ultrasound (LUS) was performed within 24 h of hospital care and analyzed using the Lung Ultrasound Combined Score (LUCS) based on the ultrasound patterns and their extent. Regression models combining LUCS (using eight, six, or four lung areas) with age and clinical scale were created. Results: A total of 90 patients were included (62 admitted to the ward, 15 to PICU), with a median age of 3.7 months. Clinical–ultrasound models with eight and six lung zones predicted hospital admission (AUC 0.89), need for oxygen therapy (AUC 0.88), and its duration (40% explanatory capacity). Models using four lung areas had lower prognostic yield. No model predicted PICU admission needs or duration. Conclusions: The ultrasound pattern and its extension combined with clinical information may be useful to predict hospital admission and oxygen requirement.

## 1. Introduction

Acute bronchiolitis (AB) is the most common lower respiratory tract infection and the leading cause of hospitalization in infants under 12 months, significantly consuming healthcare resources [1,2,3]. Pathophysiologically, there is a decrease in lung air content and an increase in fluid, altering the ventilation–perfusion ratio. These changes are visible on ultrasound, with a clinical–ultrasound correlation described by Caiulo in 2011 [4,5].

Several clinical scales exist to predict the severity of AB and/or the need for hospitalization [6,7,8,9,10]. One of the most widely used in our region is the BROSJOD (bronchiolitis score of Sant Joan de Déu), based on findings from auscultation, external signs of respiratory distress, oxygen saturation, and heart and respiratory rate [11]. However, with the expanding use of bedside ultrasound, particularly lung ultrasound (LUS), numerous studies have been conducted in recent years to evaluate its ability to predict the evolution of patients with AB in relation to various variables [12,13,14,15,16,17,18,19,20,21].

Initial studies found a relationship between the findings visualized in the LUS and variables such as clinical severity, hospital admission, admission to the pediatric intensive care unit (PICU), or the need for respiratory support [4,12,14,15,16,17,18,19,20,21]. Some of these studies also designed scores to assess the predictive capacity of LUS [4,14,15,17,18], and the most recent studies have focused on the development of models that combine the use of ultrasound with clinical variables [12,21,22].

The primary aim of this study was to develop a clinical sonographic model to predict the clinical course of children with AB in an early manner. The secondary objective was to analyze whether using ultrasound data from the anterior or antero-lateral areas may provide similar prognosis ability, similarly to studies conducted in an adult population comparing the efficacy of various protocols covering different lung areas [23,24,25].

## 2. Materials and Methods

A prospective study was conducted between December 2018 and March 2020, including infants under 12 months diagnosed with AB. AB was considered when patients had an upper airway infection in the previous 1–3 days, followed by persistent cough, increased labored breathing, and wheezes and/or crackles on auscultation [1,2] regardless of whether it was the first episode or if they had experienced a similar condition previously. Patients with a history of congenital heart disease, bronchopulmonary dysplasia, and suspected localized infection (pneumonia) were excluded.

This study was approved by the Research Ethics Committee of Principado de Asturias. It was performed in two tertiary care centers in the North of Spain. After obtaining informed consent from guardians, all patients underwent LUS within the first 24 h of contact. Sampling was convenience-based, depending on the availability of the researcher and the ultrasound machine.

Demographic and clinical data, along with LUS, were collected for each patient. Patient management followed the usual protocols of each hospital, which adhere to international guidelines [1,2]: patients were admitted to the hospital ward when they had significant respiratory distress, apneas, oxygen needs (pulse oximetry saturation < 92%), or a significant decrease in intake, and to the PICU in cases of severe respiratory failure, high oxygen requirements (Sat O_2_ < 92% with FiO_2_ > 50%), and episodes of recurrent apnea with desaturation [26]. The primary variables studied were hospital admission (to the general ward and/or PICU), PICU admission, need for oxygen (via low-flow nasal cannula or while being ventilated), length of hospital stay (overall, including ward and PICU), length of PICU stay, and duration of oxygen therapy.

### 2.1. Lung Ultrasound

**Figure 1:**
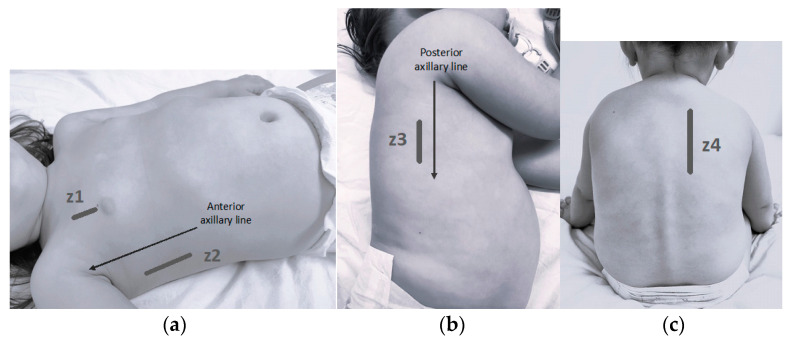
Explored lung areas (z1–z4). (**a**) z1: Anterior zone: probe in mid-clavicular line above the nipple; z2: anterolateral zone: probe between anterior and mid-axillary line, above the costal margin. (**b**) z3: Lateral-posterior zone: probe behind the posterior axillary line, above the liver. (**c**) z4: Posterior zone: probe in the paravertebral line beside the scapula.

Video clips of eight lung areas were obtained for each patient, based on previous studies [14,27] as shown in Figure 1. Image acquisition was standardized to ensure uniformity among different researchers. A linear probe was used in a cranio-caudal direction (lung preset), using two different ultrasound machines: HD7 XE (Phillips^®^, Holland, MI, USA), SonoSite M-Turbo (Fujifilm^®^, Ratingen, Germany), and Acuson NX3 Elite (Siemens^®^, Tokyo, Japan). The images were analyzed subsequently by two researchers (one with one year of experience and another with more than five) blinded to the patient’s clinical history.

Consistent with previous publications [4,13,14,15,28], each image was rated according to a system combining the visualized ultrasound pattern (normal, non-confluent B-lines, white lung, and consolidation) and its extent (1, 2, or more than 2 intercostal spaces affected), and from these initial ratings, the Lung Ultrasound Combined Score (LUCS) was developed (Table S1). Subpleural consolidations less than 1 cm in diameter with normal adjacent lung parenchyma, and the presence of ≤2 non-confluent B-lines in posterior fields (zones 3 and 4), were not considered pathological [5].

The sonographic information obtained from the scanning of all described lung areas VS. that obtained from less zones (e.g., excluding the less accessible posterior areas) was compared. LUCS was calculated with the described 8 lung areas (4 per hemithorax; Figure 1), 6 areas (anterior, anterolateral, and posterolateral -z1, z2, and z3-), and 4 areas (anterior and anterolateral -z1 and z2-), referred to as 8Z, 6Z, and 4Z, respectively.

### 2.2. Statistical Analysis

The statistical analysis was performed using the R program (R Development Core Team), version 3.6.3. Descriptive analysis was conducted, providing relative and absolute frequency distributions for qualitative variables, and measures of position and dispersion for quantitative variables. Relationships between qualitative variables were examined using the Chi-square or Fisher test, depending on the fulfillment of expected frequencies. The evaluation of different quantitative variables as tests to diagnose admission or oxygen therapy requirement was performed by calculating sensitivity, specificity, predictive values, and likelihood ratios, both positive and negative, establishing an optimal cutoff point calculated from the Youden index. The area under the receiver operating characteristic (ROC) curve (AUC) was also calculated.

Binary logistic regression models were constructed considering hospital admission, the need for oxygen therapy, and PICU admission as the main outcomes, and the LUCS ultrasound score, age, and the BROSJOD score as independent variables. First, univariate models were developed, and then multivariate models, calculating the odds ratio (OR) of each coefficient, its 95% confidence interval (CI), and the associated *p*-value. Nagelkerke’s R2, AUC, and Akaike Information Criterion (AIC) were calculated as measures of model fit. Linear models with the same independent variables were also developed to predict the duration of hospital stay, days of oxygen therapy, and PICU stay, using AIC and adjusted R2 as measures of model fit.

The degree of concordance between researchers was analyzed using the intraclass correlation coefficient (ICC).

## 3. Results

### 3.1. Sample Description

**Figure 2:**
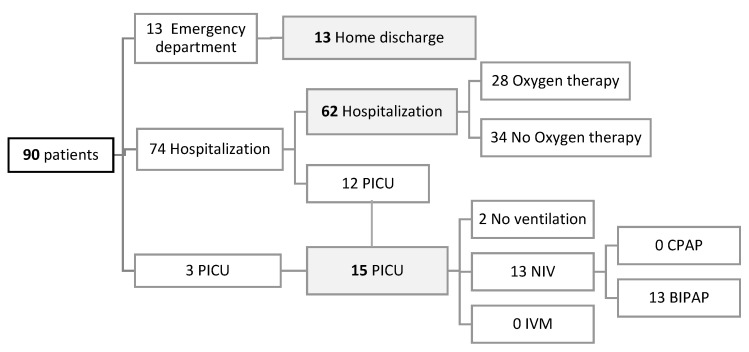
Patient flow diagram. PICU: pediatric intensive care unit. NIV: non-invasive ventilation. IMV: invasive mechanical ventilation. CPAP: continuous positive airway pressure. BIPAP: bilevel positive airway pressure.

A total of 90 patients were included, 61 from hospital A and 29 from hospital B. The median age of the individuals was 3.7 months (interquartile range—IQR—1.8–6.7 months); 62 required hospitalization and 15 required PICU admission. The sample characteristics, patient evolution, and outcomes are shown in Table 1 and Figure 2.

There were no statistically significant differences between the sample groups from the hospitals regarding history, patients’ characteristics, evolution, PICU stay, or connection to oxygen or mechanical ventilation. Differences were only found in hospital stay duration and the rate of individuals requiring oxygen therapy (37.7% in one center and 69% in another), although both of them used an oxygen saturation criterion of <92% on pulse oximetry for its application (Table S2). In terms of care requirements (need for hospitalization, need for oxygen therapy, length of hospital stay, length of PICU stay, or duration of oxygen therapy), no significant differences were observed between patients with RSV and those infected with other viruses.

### 3.2. Ultrasound Findings

Ultrasound was performed at a median of 16 h after admission (IQR 12–22). The median BROSJOD score at the time of ultrasound examination was 6 (IQR 4–7), consistent with moderate bronchiolitis. The ICC between researchers was 0.924 (95% CI 0.886–0.949).

#### 3.2.1. LUCS Relation with Outcome Variables

In all patients requiring oxygen therapy and hospitalization, the LUCS scores were significantly higher regardless of whether 8Z, 6Z, or 4Z was used. For PICU admission, significantly higher scores were obtained only with 8Z and 6Z, not with 4Z (Table S3).

#### 3.2.2. Accuracy of LUCS

The LUCS for the three main outcomes (oxygen requirement, hospital admission, and PICU admission), showed AUC values between 0.72 and 0.76 when applied to 8Z: 0.729 (95% CI 0.531–0.896; *p =* 0.008); 0.724 (95% CI 0.617–0.831; *p* < 0.001); and 0.750 (95% CI 0.615–0.886; *p* = 0.002), respectively, and 6Z: 0.764 (95% CI 0.625–0.902; *p =* 0.002); 0.730 (95% CI 0.626–0.833; *p* < 0.001); and 0.719 (95% CI 0.601–0.838; *p* = 0.014), respectively. When using 4 zones, AUC values were acceptable for hospital admission (0.698; 95% CI 0.559–0.836; *p* = 0.019) and oxygen requirement (0.674; 95% CI 0.565–0.783; *p =* 0.003), but lost significance for predicting PICU admission (0.606; 95% CI 0.447–0.765; *p* = 0.182) (Table S4).

#### 3.2.3. Logistic Regression Models

The clinical–ultrasound models developed from the 3 LUCS versions (8Z, 6Z, and 4Z, with 8, 6, and 4 lung areas, respectively) along with age and the BROSJOD clinical score, yielded the following results for the different main variables.

- Hospital Admission: LUCS reached significant values within the 8Z and 6Z models (*p* = 0.029 and *p* = 0.045, respectively). The AUCs of the models were 0.899 and 0.889, and the explanatory power (R2) was 47% (Table S6). A score > 3 in the *LUCS* 8Z would increase the risk of requiring hospital admission by more than 5 times. The 4Z option did not yield significant results (Table 2).
- Oxygen Therapy: Significant values were obtained for the score in all its versions (*p* = 0.001 for 8Z and 6Z, and *p* = 0.021 for 4Z) with AUC > 0.85 and R2 up to 55% exploring 6 zones (Table S6). In the LUCS 8Z, a score >6 would increase the risk of requiring oxygen by almost 7 times, and more than 2 points in the LUCS 4Z would increase it by 4 times (Table 2).
- PICU Admission: The LUCS score did not reach a significant value in any extension (8Z, 6Z, or 4Z) (Table 2).

#### 3.2.4. Linear Regression Models

Similar to logistic regression, linear models were constructed to predict the duration of hospital stay, PICU stay, and days of oxygen therapy (Tables S5 and S6):

- Hospital Stay: LUCS was significant within the 8 and 6 zone models (*p* = 0.001). A LUCS score > 3 or 2 (depending on whether it is 8Z or 6Z) would increase the hospital stay by almost 2 days, with an R2 for both models around 45%. The 4Z model did not reach statistical significance (*p =* 0.082).
- Oxygen Therapy Duration: Significant results were obtained only with the 8Z option (*p* < 0.001), although the results with 6 zones were close to reaching significance (*p* = 0.051).
- PICU Stay: No statistical significance was reached for the models in 8Z, 6Z, or 4Z.

## 4. Discussion

The clinical–ultrasound model developed seems to be able to predict the need for hospital admission and oxygen therapy, as well as their duration, in infants with AB. While other authors have developed scores based on ultrasound or clinical–ultrasound parameters to predict hospital admission, PICU admission, or the need for respiratory support [12,14,15,17,19,20,21,22], no studies have simultaneously evaluated these three main outcomes (hospital admission, oxygen therapy, PICU admission) or their duration.

Additionally, to our knowledge, this is the first study in the pediatric population comparing a single score (LUCS) including different number of lung areas (8Z, 6Z, and 4Z). In this regard, it appears that the use of six lung areas (omitting the paravertebral region) yields similar results to exploring the entire lung (8Z), while the performance of only four areas is lower. This finding suggests that exploring the paravertebral zone could be avoided, thus significantly facilitating exploration in critical patients. This is consistent with previous authors’ statements, since the visualization of alterations in posterior fields may largely be due to the effect of gravity after extended periods in a supine position [12,19,29], and with Pisani’s findings in adults with acute respiratory distress [23]. Ingelse, who evaluated mechanically ventilated AB patients, did not effectively assess these posterior regions [30], and the LUSCAB score developed by Bueno-Campaña only considered B-lines in the anterior thorax [12,21]. However, other studies have found that posterior zone involvement is most related to poor AB outcomes [22].

Regarding the predictive capacity of the LUCS alone, ROC analysis for the three main variables studied showed acceptable AUC values (>0.72) for the 8Z and 6Z options; exploring four areas offered similar results but only for hospital admission or oxygen therapy requirement. Gori obtained AUC values of 0.76 and 0.73 with their ultrasound scales to predict the clinical severity of AB [19]; however, they did not study their relationship with resource utilization.

The predictive capacity of the clinical–ultrasound logistic regression models for hospital admission and oxygen therapy was greater than that of the ultrasound score alone (AUC > 0.85 and explanatory power around 50% with the 8Z and 6Z models) and superior to that obtained by Hernández-Villarroel (AUC of 0.64 and 0.69), who also developed a clinical–ultrasound model [21]. Linear models were also useful for predicting days of hospital admission and oxygen therapy (although only with 8Z). None of the models (8Z, 6Z, and 4Z) were capable of predicting the need for PICU admission or its duration, which may be due to the small sample size (only 17% were admitted to this unit).

Our study has several limitations. First, there is the possibility of some selection bias due to convenience sampling, as well as differences in clinical management between the two centers. Furthermore, although image collection was highly standardized, it was carried out with different ultrasound machines. Additionally, there was a wide time margin for performing the ultrasound (from 0 to 24 h of patient contact), and the study utility relates to being applied as early as possible to patients diagnosed with BA. It is also worth noting that hospital admission does not always occur due to high scores on the clinical severity scale, but may be influenced by other factors such as inadequate oral fluid intake or the presence of apneas in the case of PICU admission, which may not be reflected in the ultrasound findings in these situations. This could partly explain the low cutoff points in some cases in the regression models (for example, >2 points on the LUCS 6Z score would increase the probability of hospital admission by nearly six times).

As strengths of this study, we should also mention the high correlation coefficient obtained between the researchers. In general, studies on point-of-care ultrasound show high levels of agreement among observers [14,17,18].

## 5. Conclusions

The present clinical–ultrasound model seems to be useful for identifying infants with AB at higher risk of requiring hospital admission or oxygen therapy. Scanning the paravertebral areas may be unnecessary.

## Figures and Tables

**Table 1:** Sample characteristics.

Patients’ Characteristics			Patients Outcome	
Age (m)		3.68 (1.80–6.65)	Discharged home	13 (14.4)
Weight (kg)		5.80 (4.71–7.70)	Hospital admission	77 (85.6)
Gestational age (w + d)		39 + 1 (37 + 5–40 + 2)	Hospital stay (d)	4 (2–6)
Birth weight (g)		3.080 (2.735–3.455)	PICU admission	15 (16.6)
Gender	Male	49 (54.43)	PICU stay (d)	4 (3–5.5)
	Female	41 (45.6)	Oxygen therapy (low flow/with NIV)	43 (47.8)
Prematurity (<37 weeks)		8 (8.9%)	Low-flow oxygen therapy only	30 (69.8)
Breastfeeding		58 (64.4)	Duration of oxygen therapy (d)	3 (1.5–5)
Daycare attendance		13 (14.4)	Need of NIV	13 (14.4)
Previous episodes of respiratory distress		22 (24.4)	Duration of NIV support (d)	2.5 (2–3)
			Need of IMV	0 (0)
Household members with respiratory symptoms		70 (77.8)		
**Nasopharyngeal Swab**				
Not collected		10 (11.1)	RSV	54 (67.5)
Collected		80 (88.9)	Adenovirus	5 (6.25)
Negative		13 (16.25)	Influenza virus	4 (5)
Coinfection		10 (12.5)	Others	4 (5)

Results are expressed as “frequency (%)” in the qualitative variables and as “median (interquartile range)” in the quantitative variables. m: months. w: weeks. d: days. PICU: pediatric intensive care unit. NIV: non-invasive ventilation. IMV: invasive mechanical ventilation.

**Table 2:** Univariate and multivariate logistic regression models.

			Cutoff Point	OR Univariate	OR Multivariate
Hospitalization	Model 8Z variables	Age (m)	>3.47	0.07 (0.00–0.36) *p* = 0.011	0.05 (0.00–0.32) *p* = 0.008
		BROSJOD	>6	7.33 (1.81–49.50) *p* = 0.013	12.71 (2.60–101.04) *p* = 0.005
		*LUCS* 8Z	>3	6.41 (1.87–25.86) *p* = 0.005	5.47 (1.28–28.60) *p* = 0.029
	Model 6Z variables	Age (m)	>3.47	0.07 (0.00–0.36) *p* = 0.011	0.06 (0.00–0.36) *p* = 0.011
		BROSJOD	>6	7.33 (1.81–49.50) *p* = 0.013	10.85 (2.27–82.31) *p* = 0.007
		*LUCS* 6Z	>2	9.10 (2.24–61.57) *p* = 0.006	5.90 (1.20–45.00) *p* = 0.045
	Model 4Z variables	Age (m)	>3.47	0.07 (0.00–0.36) *p* = 0.011	0.07 (0.00–0.42) *p* = 0.016
		BROSJOD	>6	7.33 (1.81–49.50) *p* = 0.013	11.07 (2.40–81.80) *p* = 0.005
		*LUCS* 4Z	>2	11.10 (2.04–207.16) *p* = 0.024	6.01 (0.88–121.47) *p* = 0.116
Oxygen Therapy	Model 8Z variables	Age (m)	>4.07	0.44 (0.19–1.01) *p* = 0.057	0.23 (0.06–0.73) *p* = 0.018
		BROSJOD	>6	11.02 (4.28–31.04) *p* < 0.001	15.77 (5.01–60.80) *p* < 0.001
		*LUCS* 8Z	>6	6.39 (2.60–16.86) *p* < 0.001	6.88 (2.28–23.61) *p* = 0.001
	Model 6Z variables	Age (m)	>4.07	0.44 (0.19–1.01) *p* = 0.057	0.35 (0.09–1.14) *p* = 0.093
		BROSJOD	>6	11.02 (4.28–31.04) *p* < 0.001	16.98 (5.29–67.49) *p* < 0.001
		*LUCS* 6Z	>1	16.56 (4.37–109.05) *p* < 0.001	17.45 (3.67–134.55) *p* = 0.001
	Model 4Z variables	Age (m)	>4.07	0.44 (0.19–1.01) *p* = 0.057	0.36 (0.10–1.11) *p* = 0.086
		BROSJOD	>6	11.02 (4.28–31.04) *p* < 0.001	17.22 (5.66–64.96) *p* < 0.001
		*LUCS* 4Z	>2	3.63 (1.53–8.98) *p* = 0.004	3.97 (1.29–13.91) *p* = 0.021
Picu Admission	Model 8Z variables	Age (m)	>3.37	0.10 (0.02–0.41) *p* = 0.004	0.03 (0.00–0.22) *p* = 0.005
		BROSJOD	>8	38.86 (9.83–205.02) *p* < 0.001	94.99 (13.22–2086.29) *p* < 0.001
		*LUCS* 8Z	>8	5.84 (1.80–22.86) *p* = 0.005	3.23 (0.52–26.56) *p* = 0.220
	Model 6Z variables	Age (m)	>3.37	0.10 (0.02–0.41) *p* = 0.004	0.03 (0.00–0.28) *p* = 0.008
		BROSJOD	>8	38.86 (9.83–205.02) *p* < 0.001	101.08 (14.33–2197.85) *p* < 0.001
		*LUCS* 6Z	>3	6.00 (1.73–27.98) *p* = 0.009	2.95 (0.46–26.36) *p* = 0.273
	Model 4Z variables	Age (m)	>3.37	0.10 (0.02–0.41) *p* = 0.004	0.03 (0.00–0.26) *p* = 0.007
		BROSJOD	>8	38.86 (9.83–205.02) *p* < 0.001	114.50 (16.64–2453.94) *p* < 0.001
		*LUCS* 4Z	>2	2.38 (0.78–7.77) *p* = 0.134	1.27 (0.19–9.29) *p* = 0.803

The models are constructed based on the LUCS (with its 3 versions depending on pulmonary areas explored—8Z, 6Z, and 4Z) in conjunction with age and the clinical scale. 8Z: 8 pulmonary zones. 6Z: 6 pulmonary zones. 4Z: 4 pulmonary zones. m: months. OR: odds ratio. PICU: pediatric intensive care unit. BROSJOD: Sant Joan de Déu Hospital bronchiolitis severity clinical score.

## Data Availability

The datasets used and/or analyzed during the current study are available from the corresponding author on reasonable request due to privacy and ethical restrictions.

## Supplementary Materials

The following supporting information can be downloaded at: https://www.mdpi.com/article/10.3390/children11080987/s1. Table S1: Lung Ultrasound Combined Score (LUCS). Table S2: Comparison of the different variables between the hospital centers. Table S3: Scores obtained from the different scoring systems grouped according to the need for hospitalization, oxygen therapy, and admission to the PICU or not. Table S4: Accuracy of the ultrasound score in relation to hospital admission, need for oxygen therapy, and admission to the PICU. Table S5: Linear models for predicting the duration of hospital stay, oxygen requirement, and PICU stay. Table S6: Goodness-of-fit tests applied to the developed logistic and linear regression models.
