# Supplementary material for: Clinical–Ultrasound Model to Predict the Clinical Course in Bronchiolitis
Source: Children (Basel). 2024 Aug 14;11(8):987. doi: 10.3390/children11080987 (PMC11352824; doi:10.3390/children11080987)
Supplement: Supplementary file 1 [file children-11-00987-s001.zip › children-3121435-supplementary.pdf]

SUPPLEMENTARY MATERIALS

Table S1. Lung Ultrasound Combined Score (*LUCS*).

		Points
**Pattern**	Normal aeration pattern	0
	B-lines (confluent/non-confluent)	1
	White lung	2
	Consolidation	3
**Extension**	Findings limited to 1 intercostal space	0
	Findings in 2 intercostal spaces	1
	Findings in more than 2 intercostal spaces	2

The LUCS ultrasound score is calculated by summing the scores obtained in each pulmonary area (8, 6, or 4 areas depending on the options studied). Each assessed pulmonary zone has a score resulting from the sum of the visualised ultrasound pattern and its extension.

Table S2. Comparison of the different variables between the hospital centres. Relationships between quantitative variables were examined using the Student's t-test and between qualitative variables the Chi-square test.

	**Centre A**		**Centre B**		***p***
	**n**	**Mean (SD)**	**n**	**Mean (SD)**	
Age (m)	61	4.7 (3.1)	29	4.0 (2.8)	0.35
Weight (kg)	61	6.4 (1.8)	29	5.8 (1.9)	0.17
Gestational age (w+d)	61	38.8 (2.0)	29	39.2 (2.2)	0.40
Hospital stay (d)	54	3.89 (2.74)	23	5.47 (2.99)	0.04
PICU stay (d)	13	4.25 (1.4)	2	5.0	0.61
Duration of oxygen therapy (d)	23	3.57 (2.54)	20	4.11 (2.95)	0.53
Duration of NIV (d)	11	2.3 (1.1)	2	3.0	0.54
	n	%	n	%	*p*
Gender male	34	55.74	15	51.72	0.72
Fever	21	34.43	13	44.83	0.25
Need for hospital admission	54	88.5	23	79.3	0.25
Need for oxygen therapy	23	37.7	20	69	0.01
Need for PICU admission	13	21.3	2	6.9	0.09

m: months. w: weeks. d: days. PICU: paediatric intensive care unit. NIV: Non-invasive ventilation. SD: standard deviation.

Table S3. Scores obtained from the different scoring systems grouped according to the need for hospitalization, oxygen therapy, and admission to the PICU or not.

LUCS		**HOSPITALIZATION**			**OXYGEN THERAPY**			**PICU ADMISSION**		
		**n**	**Median (p25-p75)**	***p***	**n**	**Mean (SD)**	***p***	**n**	**Mean (SD)**	***p***
**8Z**	No	13	1 (0-4)	0.008	47	5.02 (5.93)	0.001	75	6.12 (6.0)	0.001
	Yes	77	6 (2-11)		43	9.30 (6.17)		15	11.80 (6.33)	
**6Z**	No	13	0 (0-1)	0.002	47	2.38 (3.29)	0.002	75	3.13 (3.83)	0.011
	Yes	77	3 (1-6)		43	4.95 (4.31)		15	6.00 (4.14)	
**4Z**	No	13	1 (0-4)	0.008	47	1.45 (2.39)	0.017	75	1.97 (2.70)	0.365
	Yes	77	6 (2-11)		43	2.79 (2.84)		15	2.67 (2.64)	

The results are presented as mean (SD) or median (p25-p75). 8Z: 8 pulmonary zones. 6Z: 6 pulmonary zones. 4Z: 4 pulmonary zones. SD: standard deviation. PICU: paediatric intensive care unit.

Table S4. Accuracy of the ultrasound score in relation to hospital admission, need for oxygen therapy, and admission to the PICU.

	LUCS	Cut-off point	Se (%)	Sp (%)	PPV (%)	NPV (%)	LR +	LR -	AUC (CI)	*p* value
**HOSPITALIZATION**	**8Z**	3	74.03	69.23	93.44	31.03	2.41	0.42	0.729 (0.531-0.896)	0.008
	**6Z**	2	62.34	84.62	96	27.5	4.05	0.25	0.764 (0.625-0.902)	0.002
	**4Z**	2	48.05	92.31	97.37	23.08	6.25	0.16	0.698 (0.559-0.836)	0.019
**OXYGEN THERAPY**	**8Z**	6	76.74	65.96	67.35	75.61	2.25	0.44	0.724 (0.617-0.831)	<0.001
	**6Z**	1	95.35	44.68	61.19	91.3	1.72	0.58	0.730 (0.626-0.833)	<0.001
	**4Z**	2	58.14	72.34	65.79	65.38	2.10	0.48	0.674 (0.565-0.783)	0.003
**PICU ADMISSION**	**8Z**	8	73.33	68	31.43	92.73	2.29	0.44	0.750 (0.615-0.886)	0.002
	**6Z**	3	83.33	58.67	24.39	95.65	2.02	0.50	0.719 (0.601-0.838)	0.014
	**4Z**	2	60	62.67	24.32	88.68	1.61	0.62	0.606 (0.447-0.765)	0.182

8Z: 8 pulmonary zones. 6Z: 6 pulmonary zones. 4Z: 4 pulmonary zones. Se: sensitivity. Sp: specificity. PPV: positive predictive value. NPV: negative predictive value. LR+: positive likelihood ratio. LR-: negative likelihood ratio. AUC: area under the curve. CI: Confidence interval.

Table S5. Linear models for predicting the duration of hospital stay, oxygen requirement, and PICU stay.

			Cut-off point	Univariate Coefficient	Multivariate Coefficient
HOSPITAL STAY	**Model 8Z** variables	Age (m)	>3.47	-1.56 (-2.84 to -0.27) p=0.019	-1.48 (-2.51 to -0.46) p=0.005
		BROSJOD	>6	3.31 (2.19 to 4.43) p<0.001	3.35 (2.36 to 4.35) p<0.001
		LUCS 8Z	>3	2.45 (1.15 to 3.75) p<0.001	1.83 (0.75 to 2.90) p=0.001
	**Model 6Z** variables	Age (m)	>3.47	-1.56 (-2.84 to -0.27) p=0.019	-1.35 (-2.39 to -0.32) p=0.011
		BROSJOD	>6	3.31 (2.19 to 4.43) p<0.001	3.22 (2.22 to 4.21) p<0.001
		LUCS 6Z	>2	2.66 (1.46 to 3.86) p<0.001	1.84 (0.79 to 2.88) p=0.001
	**Model 4Z** variables	Age (m)	>3.47	-1.56 (-2.84 to -0.27) p=0.019	-1.56 (-2.68 to -0.44) p=0.007
		BROSJOD	>6	3.31 (2.19 to 4.43) p<0.001	3.42 (2.38 to 4.46) p<0.001
		LUCS 4Z	>2	1.81 (0.50 to 3.13) p=0.008	1.02 (-0.13 to 2.17) p=0.082
OXYGEN THERAPY	**Model 8Z** variables	Age (m)	>4.07	-1.03 (-2.14 to 0.09) p=0.071	-1.03 (-1.92 to -0.14) p=0.024
		BROSJOD	>6	2.71 (1.73 to 3.69) p<0.001	2.39 (1.48 to 3.29) p<0.001
		LUCS 8Z	>6	2.35 (1.32 to 3.37. p<0.001	1.74 (0.83 to 2.65) p<0.001
	**Model 6Z** variables	Age (m)	>4.07	-1.12 (-2.25 to 0) p=0.050	-1.01 (-2.00 to -0.03) p=0.044
		BROSJOD	>6	2.66 (1.66 to 3.66) p<0.001	2.54 (1.57 to 3.51) p<0.001
		LUCS 6Z	>1	1.93 (0.69 to 3.17) p=0.003	1.13 (-0.00 to 2.26) p=0.051
	**Model 4Z** varia-	Age (m)	>4.07	-1.12 (-2.25 to 0.00) p=0.050	-1.01 (-2.02 to 0.01) p=0.051
		BROSJOD	>6	2.66 (1.66 to 3.66) p<0.001	2.69 (1.73 to 3.65) p<0.001

		LUCS 4Z	>2	1.27 (0.13 to 2.42) p=0.029	0.85 (-0.18 to 1.88) p=0.104
**PICU STAY**	**Model 8Z** variables	Age (m)	>3.37	0.23 (-2.09 to 2.55) p=0.833	-0.33 (-2.51 to 1.84) p=0.737
		BROSJOD	>8	2.14 (0.29 a 3.98) p=0.027	2.33 (0.16 to 4.51) p=0.038
		LUCS 8Z	>8	-0.03 (-2.02 to 1.96) p=0.971	0.50 (-1.39 to 2.39) p=0.563
	**Model 6Z** variables	Age (m)	>3.37	0.23 (-2.09 to 2.55) p=0.833	-0.21 (-2.39 to 1.96) p=0.829
		BROSJOD	>8	2.14 (0.29 to 3.98) p=0.027	2.21 (0.04 to 4.39) p=0.047
		LUCS 6Z	>3	-0.23 (-2.55 to 2.09) p=0.833	0.21 (-1.96 to 2.39) p=0.829
	**Model 4Z** variables	Age (m)	>3.37	0.23 (-2.09 to 2.55) p=0.833	-0.18 (-2.29 to 1.92) p=0.848
		BROSJOD	>8	2.14 (0.29 to 3.98) p=0.027	2.03 (-0.20 to 4.26) p=0.069
		LUCS 4Z	>2	-0.80 (-2.44 to 0.84) p=0.306	-0.31 (-1.94 to 1.33) p=0.681

The models are constructed based on the *LUCS* (with its three versions depending on the extent of pulmonary coverage - 8Z, 6Z, and 4Z) in conjunction with age and the clinical scale. 8Z: 8 pulmonary zones. 6Z: 6 pulmonary zones. 4Z: 4 pulmonary zones. m: months. PICU: paediatric intensive care unit. BROSJOD: Sant Joan de Déu Hospital bronchiolitis severity clinical score.

Table S6. Goodness-of-fit tests applied to the developed logistic and linear regression models.

**Logistic regression**	**Hospitalization**			**Oxygen therapy**			**PICU admission**		
	R^2 (%)	AUC	AIC	R^2 (%)	AUC	AIC	R^2 (%)	AUC	AIC
LUCS 8Z	47.87	0.899	54.12	53.6	0.881	86.35	68.31	0.95	42.27
LUCS 6Z	47.25	0.889	54.54	55.87	0.885	83.75	67.99	0.94	42.56
LUCS 4Z	42.87	0.894	59.51	47.74	0.853	92.72	66.61	0.933	43.79

**Linear regression**	**Hospital stay**		**Oxygen therapy duration**		**PICU stay**	
	R^2_{adj} (%)	AIC	R^2_{adj} (%)	AIC	R^2_{adj} (%)	AIC
LUCS 8Z	44.90	387.01	39.40	379.13	19.75	46.38
LUCS 6Z	45.40	386.24	31.32	391.88	17.00	46.82
LUCS 4Z	39.73	394.63	30.34	393.11	18.18	46.63

R^2: Nagelkerke's R-squared. AUC: Area Under the Curve. AIC: Akaike Information Criterion. R^2_{adj}: Adjusted R-squared.
